# Two-Dimensional Tellurium: Progress, Challenges, and Prospects

**DOI:** 10.1007/s40820-020-00427-z

**Published:** 2020-04-21

**Authors:** Zhe Shi, Rui Cao, Karim Khan, Ayesha Khan Tareen, Xiaosong Liu, Weiyuan Liang, Ye Zhang, Chunyang Ma, Zhinan Guo, Xiaoling Luo, Han Zhang

**Affiliations:** 1 Institute of Microscale Optoelectronics, International Collaborative Laboratory of 2D Materials for Optoelectronics Science and Technology, Key Laboratory of Optoelectronic Devices and Systems of Ministry of Education and Guangdong Province, College of Physics and Optoelectronic Engineering, Shenzhen Key Laboratory of Micro-Nano Photonic Information Technology, Guangdong Laboratory of Artificial Intelligence and Digital Economy (SZ), Shenzhen University, Shenzhen, 518060 Guangdong People’s Republic of China; 2 Department of Ophthalmology, Shenzhen People’s Hospital, Second Clinical Medical College of Jinan University, First Affiliated Hospital of Southern University of Science and Technology, Shenzhen, 518020 Guangdong People’s Republic of China; 3 School of Electrical Engineering and Intelligentization, Dongguan University of Technology, Dongguan, 523808 Guangdong People’s Republic of China

**Keywords:** 2D materials, Tellurium, Photodetectors, Solar cells, Energy harvesting, Logic gate and circuits

## Abstract

Since the successful fabrication of two-dimensional (2D) tellurium (Te) in 2017, its fascinating properties including a thickness dependence bandgap, environmental stability, piezoelectric effect, high carrier mobility, and photoresponse among others show great potential for various applications. These include photodetectors, field-effect transistors, piezoelectric devices, modulators, and energy harvesting devices. However, as a new member of the 2D material family, much less known is about 2D Te compared to other 2D materials. Motivated by this lack of knowledge, we review the recent progress of research into 2D Te nanoflakes. Firstly, we introduce the background and motivation of this review. Then, the crystal structures and synthesis methods are presented, followed by an introduction to their physical properties and applications. Finally, the challenges and further development directions are summarized. We believe that milestone investigations of 2D Te nanoflakes will emerge soon, which will bring about great industrial revelations in 2D materials-based nanodevice commercialization.

## Highlights

- Physical Properties of the two-dimensional tellurium were discussed in detail, including electrical properties, optical properties, thermoelectric properties, and outstanding environmental stability.
- Emerging applications based on atomically thin tellurene flakes were presented, such as photodetector, transistors, piezoelectric device, modulator, and energy harvesting devices.
- The challenges encountered and prospects were presented.

## Graphical abstract

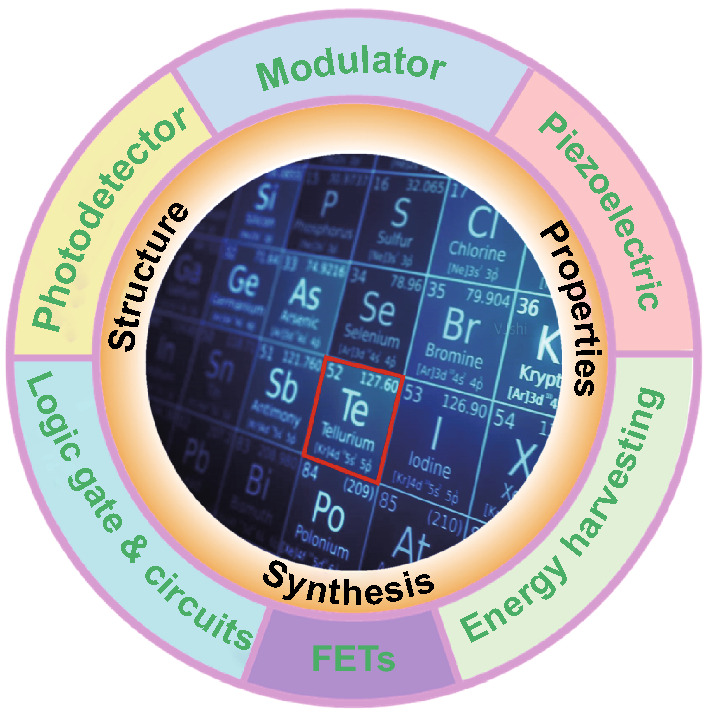

## Introduction

As one of the chalcogens (group-VI materials), tellurium (Te) is well known as a p-type semiconductor with a bandgap of 0.35 eV at room temperature and possesses a wealth of intriguing properties [1] such as photoconductivity [2], thermoelectricity [3], and piezoelectricity [4]. Since atomically thin graphene flakes were discovered in 2004 [5, 6], two-dimensional (2D) materials have triggered intensive research interest for the fabrication of nanodevices on an industrial scale [7–15]. However, the development of 2D materials faces significant challenges, such as the zero bandgap of graphene [16, 17], the environmental instability of black phosphorus (BP) [18–22], the low current mobility of transition metal dichalcogenides (TMDCs) [23], and the lack of large-scale and efficient synthesis methods. In 2017, 2D nanoflakes of Te were successfully fabricated [24], which possess superior properties compared to other existing 2D materials, including excellent environmental stability, better oxidation and hydration catalytic activity, a tunable bandgap, improved thermoelectric, and nonlinear optical responses, and a high carrier mobility (~ 10^3^ cm^2^ V^−1^ s^−1^) at room temperature [25]. These properties are favorable for fundamental research and practical applications, such as high-performance photodetectors [26], field-effect transistors (FETs), and modulators. In addition, 2D Te nanoflakes possess unique helical chain structures [27], which give rise to their high carrier mobility and strong in-plane anisotropic properties. The flexible mechanical properties and structural symmetry-breaking of the 2D Te nanoflakes provide a large in-plane piezoelectric coefficient, which enables it to be a potential material for piezoelectric devices. Moreover, 2D Te nanoflakes currently possess the lowest lattice thermal conductivity among the family of known 2D single-element materials, which exhibit extraordinary topological properties [28, 29]. However, as a new member of the monoelemental 2D materials family, less is known about it compared to graphene [11, 16, 17, 30–34], BP [18, 35–41], TMDCs [42–46], and other more commonly used 2D materials [47–52]. Much more work is needed to further investigate the potential properties, schemes to control the morphology during the synthesis process, carrier dynamics, transport mechanisms, and nanodevice applications of 2D Te nanoflakes. In this regard, a detailed and comprehensive understanding of 2D Te nanoflakes is necessary for the further development of 2D Te research and technology. Inspired by this, we have summarized the recent progress in the field of 2D Te nanoflakes. In this review, we first briefly summarize the synthesis method, structure and properties of 2D Te nanoflakes. Then, we highlight some recently demonstrated progress based on 2D Te, including photodetectors, FETs, piezoelectric devices, and modulators. A consideration of prospective challenges and future research into 2D Te nanoflakes is also presented in this review.

## Structure and Synthesis Methods for 2D Te Nanostructures

As mentioned above, due to the excellent performance, the 2D Te nanostructures play a key role in many applications, such as electronics, sensors, optoelectronic devices, and energy devices. In the past two decades, numerous studies have mainly focused on the synthesis method for zero- and one-dimensional (0D and 1D) Te nanostructures [53–62]. However, for 2D Te nanostructures, relatively little is known compared to the 0D and 1D Te structural properties and synthesis methods. Therefore, in this section, we summarize and highlight some recent representative investigations regarding the structure of 2D Te. Then, we focus on the synthesis of 2D Te nanostructures, including molecular beam epitaxy (MBE), physical vapor deposition (PVD), solution synthesis, liquid-phase exfoliation (LPE), and thermal evaporation.

### Structure

**Fig. 1:**
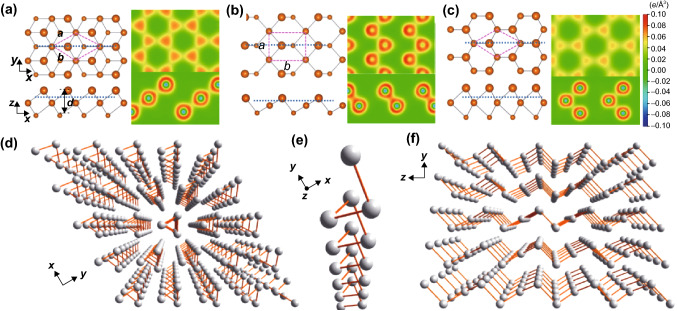
**a**–**c** Structures of α-, β-, and γ-Te phases. Adapted with permission from [63]. Copyright 2017, American Physical Society. **d**–**f** Crystal structure of Te viewed from the *z* axis, as a single-molecule chain, and viewed from the *x* axis. Adapted with permission from [26]. Copyright 2018, American Chemical Society

Through a combination of first-principles calculations and experiments, Zhu et al. [63] discovered that 2D Te (a.k.a. tellurene) possesses three phases, (α-, γ-Te) and tetragonal (β-Te) structures, as shown in Fig. 1a–c. The formation mechanism was found to be inherently rooted in the multivalent nature of Te. The α- and γ-Te phases showed a three- and sixfold coordination structure, respectively. However, the β-Te phase exhibited a mixture of three- and fourfold coordination structures; these findings suggested that Te possesses multiple bonding configurations. Subsequently, Qiao et al. [64] reported a similar investigation of the structure of few-layer Te. The crystal structure of the α-phase was found to consist of parallel helical Te chains with three Te atoms were included in each repeating unit. The β-phase can be achieved by further decreasing the thickness of the α-phase Te to a monolayer and the structure of the β-Te proved to be in accordance with BP. Additionally, no soft phonon modes were observed for monolayer β-Te, and a cohesive energy of 2.567 eV atom^−1^ indicated that the kinetics of single-layer β-Te is relative more stable [24]. Notably, Te is composed of atomic chains in a triangular helix, which are stacked together via van der Waals forces in a hexagonal array and possess a 1D crystal structure rather than a layered 2D van der Waals structure (Fig. 1d). Furthermore, Te atoms form covalent bonds to only the two closest neighboring Te atoms in the helical chain (Fig. 1e), which is in sharp contrast to the structure of other traditional 2D materials like grapheme, BP, and TMDCs that possess layered structures with strong chemical bonds within the layer. When viewed along the *x*-axis, the zigzag layers are seen to be stacked together via van der Waals forces to form a 3D structure (Fig. 1f) [25, 26].

### Synthesis Method

For BP, high-quality and large-area 2D flakes are difficult to synthesize; in contrast, the 2D Te nanoflakes can be directly synthesized via multiple facile methods [65]. In this section, four commonly employed synthesis techniques to produce 2D Te are introduced, namely PVD, MBE, solution synthesis, LPE, and thermal evaporation.

#### Physical Vapor Deposition

**Fig. 2:**
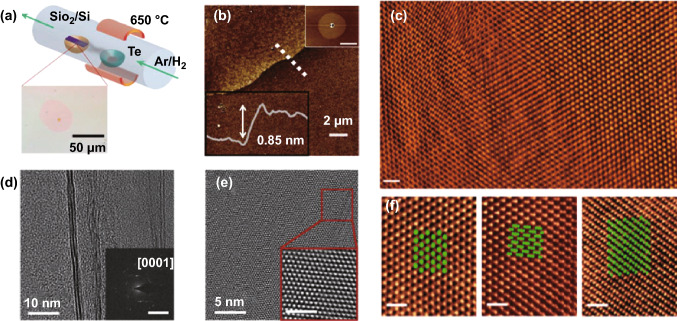
Ultrathin Te flakes synthesized by PVD. **a** Schematic of the experimental setup. **b** Atomic force microscopy (AFM) image of the edge of a Te flake including a profile taken along the dotted line showing the thickness of the flake. **c** High-angle annular dark-field scanning transmission electron microscopy (HAADF–STEM) image of Te flakes showing the large-scale uniformity. **d**, **e** TEM images of Te flakes showing their structure measured by electron diffraction (inset of **d**). **f** Atomically resolved HAADF–STEM images of the three Te polymorphs. Adapted with permission from [66]. Copyright 2019, WILEY–VCH

The PVD synthesis method is commonly applied by heating a source reservoir to control the deposition of materials onto substrates. To produce2D materials, the PVD synthesis method generally requires a vacuum environment and high-purity sources. Recently, this method has been used by Apte et al. [66] in an investigation of polytypism in the synthesis of ultrathin Te flakes with a thickness of < 7 nm and an area of 50 μm. To gain an insight into the PVD-synthesized Te flake structures, they were compared with the theoretically predicted structures. During the synthesis process, the bulk Te was first placed on the Si/SiO_2_ substrates before being evaporated in an Ar/H_2_ environment at 650 °C. After cooling down, ultrathin Te flakes were achieved, as shown in Fig. 2. The synthesized Te flakes had a typical thickness of 0.85 nm, corresponding to three atomic layers (Fig. 2b). Transmission electron microscopy (TEM) images of the synthesized flakes are shown in Fig. 2d, e, which confirmed the hexagonal symmetry with three distinct sets of sixfold diffraction spots. Utilizing high-resolution scanning transmission electron microscope (STEM) images of the Te flakes (Fig. 2c, f), the existence of three poly types, α-, β-, and γ-Te was confirmed.

#### Molecular Beam Epitaxy

**Fig. 3:**
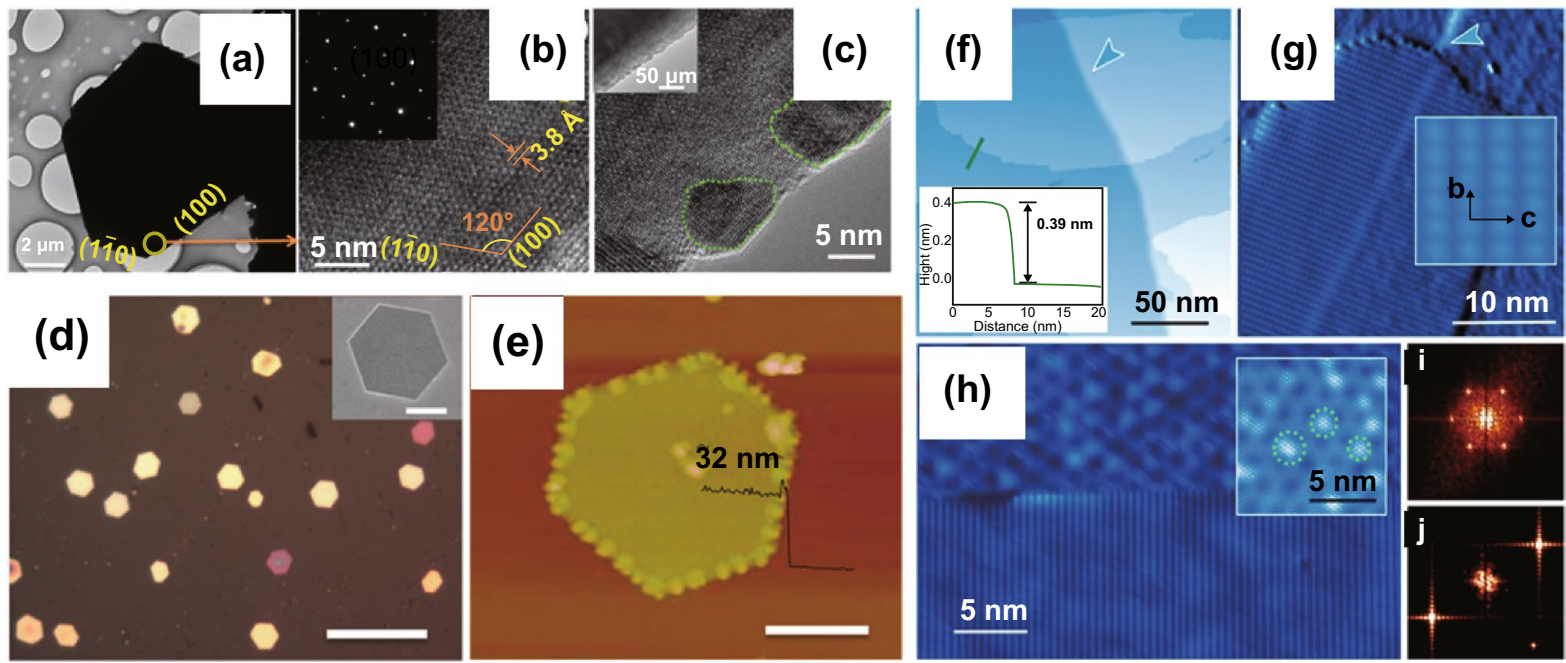
**a**, **b** TEM and high-resolution TEM (HRTEM) images of a 2D Te flake. Inset of **b** shows the electron diffraction pattern. **c** HRTEM image of the Te flake edge. **d** Optical microscope (OM) image of 2D Te flakes, scale bar = 4 μm, with inset of a single flake. **e** AFM image of 2D Te flake, scale bar = 2 μm, with the step height profile of the flake edge. Adapted with permission from [67]. Copyright 2014, American Chemical Society. **f** STM image of 2D Te grown on graphene with a step height profile of the edge. **g** High-resolution STM of monolayer Te flake. **h** STM image of the reconstructed graphene (upper and inset) and single-layer Te flake (lower). **i**, **j** Fast Fourier transforms of graphene and single-layer Te flake. Adapted with permission from [68]. Copyright 2017, American Chemical Society

In contrast to the conventional heteroepitaxy method, the van der Waals epitaxy (vdWE) synthesis method is of great interest to produce ultrathin 2D layered materials. This synthesis method can overcome the large lattice mismatch and facilitate the migration of the 2D material’s adatoms along a mica substrate surface. Additionally, the vdWE method enables over layers to be relaxed perfectly without considering the strain in the heterointerface. Recently, vdWE has been employed for the synthesis of 2D Te thin films on mica and graphene substrates [67], as shown in Fig. 3. The resulting 2D Te nanoflakes grown on the mica substrate exhibited large lateral dimensions (30–80 nm) and highly singular crystallinity, as shown in Fig. 3d, e. The chemical composition and microstructure of the Te flakes were characterized by TEM. Figure 3a–c shows the hexagonal profile of the whole sample, one corner, and an edge of the Te nanoplates, respectively. Furthermore, 2D Te flakes with mono- and few-layer thicknesses were synthesized successfully on a graphene/6H-SiC (0001) substrate, as shown in Fig. 3f–j. Using scanning tunneling microscopy (STM) measurements, the obtained Te flakes were found to be composed of parallel helical Te chains located on the surface of the graphene substrate. It can be seen from Fig. 3g, h that the lowest step height between the graphene substrate and the Te flake was approximately 0.13 nm, which confirmed that single-layer Te flakes were achieved. The fast Fourier transform (FFT) measurement showed that the Te flakes exhibited a rectangular lattice structure, which was in sharp contrast to the hexagonal symmetry of the graphene, as shown in Fig. 3i, j.

#### Solution Syntheses

**Fig. 4:**
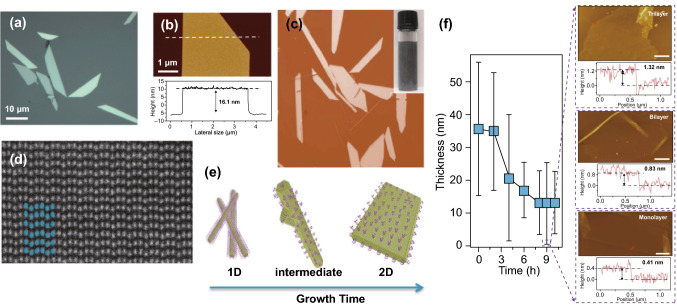
**a** Optical image of the 2D Te nanoplates. **b** AFM image of 2D Te nanoplate with height profile. Adapted with permission from [26]. Copyright 2018, American Chemical Society. **c**, **d** Optical and HAADF–STEM images of 2D Te nanoplates. **e** Morphology evolution process from 1D to 2D Te. **f** Post-growth thinning process to obtain few- and single-layer Te flakes (solution pH = 10.5). Adapted with permission from [25]. Copyright 2018, Nature Publishing Group

The production of 2D materials with large areas and high quality is essential for their further development in large-scale electronic and optoelectronics applications [69, 70]. Recently, several investigations have presented the fabrication of large-area and high-quality 2D Te nanoplates based on the solution synthesis technique [25, 26]. Figure 4a, b presents solution-synthesized environmentally stable quasi-2D Te flakes. Firstly, Na_2_TeO_3_ was dissolved into a polyvinylpyrrolidone (PVP) solution, and then, hydrazine monohydrate ammonium and hydroxide solution were added. Finally, the mixture was transferred into a Teflon-lined autoclave. After heating, cooling, and purifying processes, quasi-2D Te nanoflakes with a thickness of 10–30 nm and lateral dimensions of 10–50 μm were obtained. Additionally, the thickness of the 2D Te flakes showed a dependence on the duration of the reaction time. Subsequently, Wang et al. [25] demonstrated a substrate-free solution method to synthesize high-quality and large-scale 2D Te nanoplates. The 2D Te flakes were synthesized through reducing the concentration of the Na_2_TeO_3_ by the addition of N_2_H_4_ in an alkaline solution, in the presence of the crystal-face-blocking ligand PVP. The optical image of the obtained 2D Te solution, after the heating process, is shown in Fig. 4c. Figure 4d shows an atomically resolved HAADF–STEM image, which confirmed the threefold screw symmetry and helical chains along the [0001] direction of the 2D Te flakes. Furthermore, it was found that the morphology evolution process transitions from 1D to 2D Te, as shown in Fig. 4e, which can be attributed to the balance between kinetic and thermo dynamic mechanisms during the synthesis process. Large-area 2D Te nanoflakes with mono-, bi-, tri-, and few-layers can be obtained by tuning the pH values of the solutions, as shown in Fig. 4f.

#### Liquid-Phase Exfoliation

**Fig. 5:**
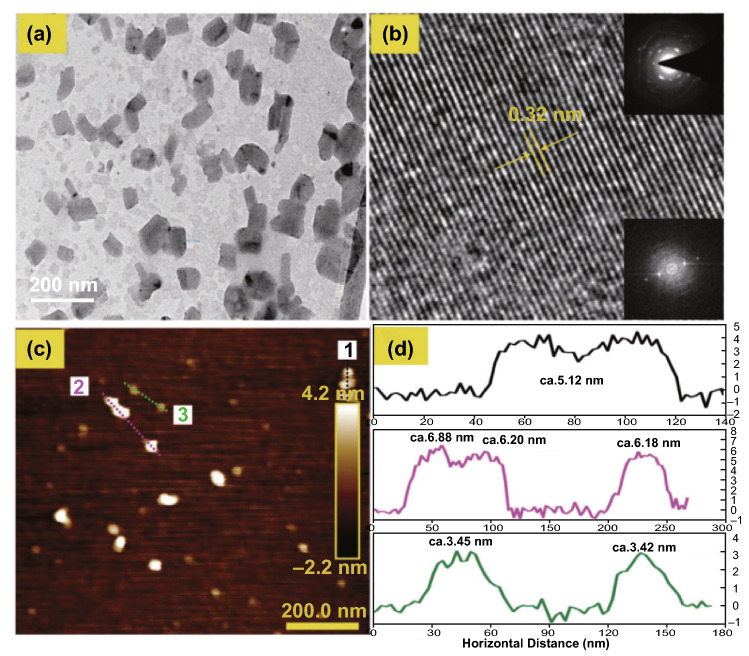
**a**, **b** TEM and HRTEM images of the 2D Te nanoflakes. Insets of **b**: SED pattern and FFT photograph of the 2D Te nanoflakes (top and bottom). **c** AFM image of the ultrathin 2D Te nanoflakes. **d** Height profiles of the 2D Te nanoflakes presented in part **c**. Adapted with permission from [75]. Copyright 2018, WILEY–VCH

The LPE technique has been an effective means of synthesizing 2D Te layered nanoarchitectures [69–74]. Generally, the exfoliation efficiency can be determined by several factors, including sonication energy, favored anisotropic characteristics, and solvent-nanoflake interactions of the bulk materials. Recently, Xie et al. [75] have reported the first production of ultrathin 2D Te nanoflakes by employing the LPE synthesis technique. Firstly, the Te powder was dissolved in IPA, and the mixture was then transferred into a plastic tube, followed by probe sonication. Finally, the 2D Te nanosheet solution was obtained by further subjecting the mixture to a bath sonication. After centrifugation and drying, ultrathin 2D Te nanoflakes were achieved with lateral dimensions ranging from 41.5 to 177.5 nm, as shown in Fig. 5a. The crystal lattice spacing was measured to be 3.2 Å, as shown in Fig. 5b. To confirm that the crystalline features of the 2D Te nanoflakes were retained during the LPE process, as elected electron diffraction (SED) pattern and FFT photograph were obtained, as shown in the inset of Fig. 5b and c, d. The thickness of the obtained 2D Te nanoflakes was measured by AFM and ranged from 3.4 ± 0.3 to 6.4 ± 0.2 nm.

#### Thermal Evaporation

**Fig. 6:**
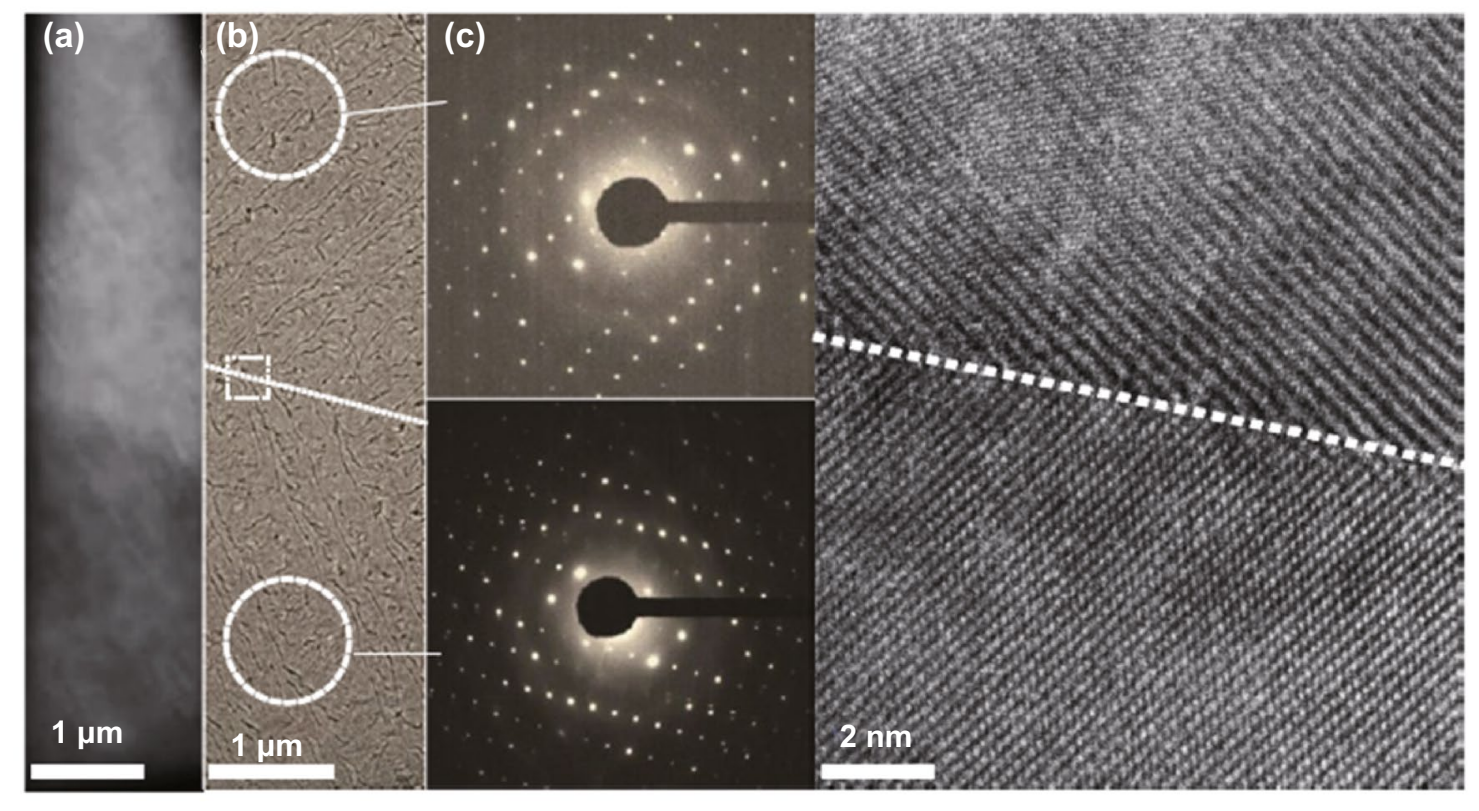
**a** Optical image of the synthesized Te nanoflakes. **b**, **c** TEM and HRTEM images of the Te nanoflakes present in **a**. Adapted with permission from [80]. Copyright 2020, Nature publishing Group

The synthesis of large-scale polycrystalline Te flakes was presented through thermal evaporation in the 1960s [76–78]. After a prolonged endeavor, Zhao et al. have recently demonstrated an exciting breakthrough in the synthesis of high-quality 2D Te thin films via this technique [79]. According to their report, Te pellets were used as the thermal vaporation source. After decreasing the pressure and temperature of the process in an Edwards Coating System, ultrathin 2D Te nanoflakes with thicknesses ranging from 4 to 53 nm were synthesized through thermal evaporation at a temperature of − 80 K. Figure 6a-c presents the optical, low-magnification TEM, and HRTEM images of the 9-nm-thick Te nanoflakes on an SiO_2_ TEM grid. Noticeably, the substrate temperature has a significant influence on the quality of the synthesized Te nanoflakes. By decreasing the substrate temperature from room temperature to − 80 K, the average area of the domains monotonically increased from zero to ~ 25 μm^2^.

## Physical Properties of 2D Te

The 2D Te nanoflakes, as a new member of the 2D materials family, have received less investigation into their physical properties. Their superior properties including a tunable direct bandgap, high carrier mobility, excellent thermoelectric performance, and stability show that 2D Te nanoflakes have great potential for electronics and photoelectronics applications. In this regard, we briefly summarize the investigations into their physical properties in this section based on first-principles calculations within ab initio molecular dynamics (MD) and density functional theory (DFT) simulations.

### Electrical Properties

**Fig. 7:**
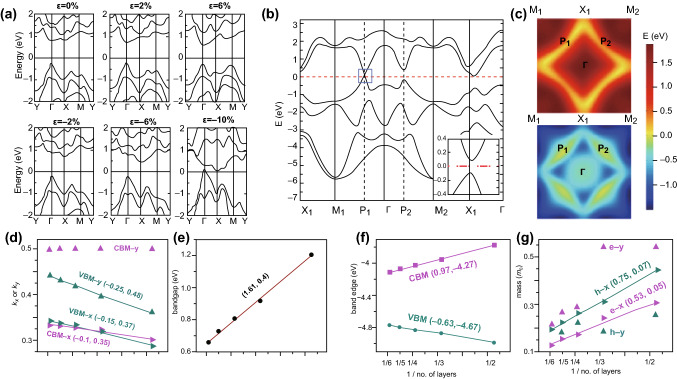
**a** Band structures of 2D Te under different biaxial strains with SOC. Adapted with permission from [82]. Copyright 2016, arXiv. **b**, **c** Band structures and outline of the bottom CB (top of **c**) and top VB band (bottom of **c**) in the first Brillouin zone. Adapted with permission from [83]. Copyright 2017, WILEY–VCH. **d** Position of the VBM and CBM. **e** Bandgaps. **f** Profile of band edge energies as the number of layers varies. **g** Effective masses of electrons and holes as the number of layers vary. Adapted with permission from [84]. Copyright 2018, American Chemical Society

Owing to the great potential of mono- and few-layer 2D Te in electronics and photoelectronics applications, the electronic band structure has recently been investigated through various theoretical calculations, including first-principles calculations based on DFT and ab initio MD simulations [81]. Zhu et al. [82] reported that monolayer 2D Te possesses a direct bandgap of 1.04 eV by using first-principles calculations. Additionally, by introducing an external strain, both the transport properties and the bandgap can be tuned, as shown in Fig. 7a. As the tensile strain was increased from 0 to 6%, the conduction band minimum (CBM) showed a gradual downshift behavior toward the Fermi level. In contrast, the valence band maximum (VBM) barely changed. Consequently, the bandgap decreased to 0.86 eV for 6% tensile strain. Xian et al. [83] presented results by first-principles calculations suggesting that 2D Te possessed a chair-like buckled structure rather than a hexagonal structure. Owing to this special structure, the 2D Te caused anisotropic band dispersions around the Fermi level, which can be explained via a generalized semi-Dirac Hamiltonian. The calculated band structure, as well as the spin–orbit coupling (SOC) of 2D Te, is shown in Fig. 7b, c. It can be seen clearly in Fig. 7a that Dirac-cone-like dispersions occurred at P_1_ in the Brillouin zone (BZ). Furthermore, in contrast to the dispersions of the group-IV 2D materials, these dispersions showed highly anisotropic behavior, as shown in Fig. 7c. Recently, Liu et al. [84] have presented work with first-principles calculations showing that along different transport directions, the isotropy of the few-layer 2D Te is related to the potential and effective mass of the charge carriers, as shown in Fig. 7d–g. More importantly, the calculated bandgaps increased as the thickness of the few-layer 2D Te decreased. The band edge energies also showed similar behavior, varying linearly with 1/n or 1/d (where n and d denote the layer number and thickness of the 2D Te nanoflakes, respectively), as shown in Fig. 7f. Additionally, the effective mass of charge carriers being transported across the chains increased linearly with 1/n or 1/d. These findings enable the evaluation of the electronic properties of 2D Te at different thicknesses.

### Optical Properties

**Fig. 8:**
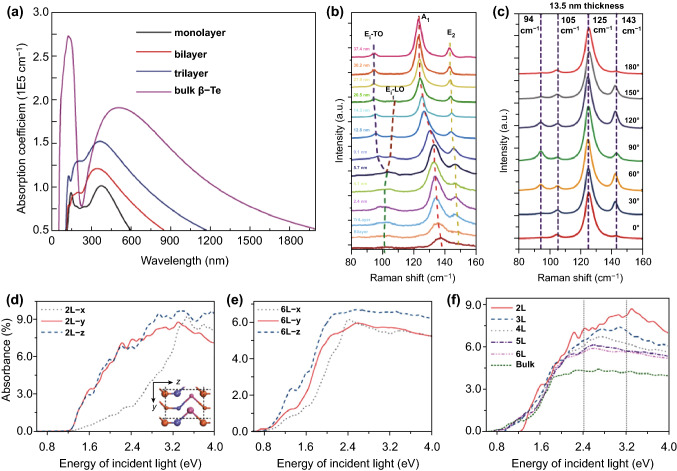
Optical properties of 2D Te. **a** Absorption coefficients of the bulk, few-layer, and monolayer β-Te. Adapted with permission from [24]. Copyright 2017, IOP Publishing. **b, c** Raman and angle-resolved Raman spectra of the 2D Te flakes. Adapted with permission from [25]. Copyright 2018, Nature Publishing Group. **d**, **e** Absorbance of 2 layer and 6-layer α-Te with the incident light polarization along the *x*-, *y*-, and *z*-direction. **f** Absorbance of incident light polarized in the direction for few-layer α- and bulk Te. Adapted with permission from [64]. Copyright 2018, Elsevier

Optical properties are another important parameter for electronics and photoelectronics applications of 2D Te, in particular for photodetectors and FET devices [85, 86]. Recently, investigations have been carried out to gain a better insight into the optical properties of 2D Te. Firstly, Wu et al. [24] reported strong light absorption in few-layer β-Te from the ultraviolet (UV) band to the visible band, as shown in Fig. 8a. For the few-layer β-Te, the calculated optical absorption coefficients showed clear layer-dependent behavior, in which the absorption coefficients decreased as the thickness of the few-layer β-Te increased. This was attributed to the thickness-dependent band dispersion and interlayer electronic hybridization; both processes were enhanced with increasing thickness. These outcomes showed that few-layer β-Te is a promising material for acousto-optic and UV–visible deflectors. As previously mentioned, 2D Te can be synthesized with large areas, which enables high-performance FETs and photodetectors based on 2D Te. Recently, Wang et al. [25] have reported an investigation on FETs based on large-scale 2D Te. In their report, thickness- and angle-dependent Raman spectra were employed to characterize the optical properties of 2D Te at room temperature, as shown in Fig. 8b, c. Three Raman active modes were observed as the thickness of the 2D Te was varied from a monolayer to 37.4 nm. Additionally, for a moderate thickness of the 2D Te flakes (less than 20.5 nm), three different Raman active modes located at approximately 92, 121, and 125 cm^−1^ were found, which is consistent with previous investigations. These findings suggested that the symmetric characteristics of the bulk Te were appropriate for its 2D morphology. Further decreasing the thickness to 9.1 nm, the E_1_ longitudinal (LO) mode appeared, and this can be attributed to the enhanced deformation potential and the weakened electro-optic effect in the 2D Te lattice. As the thickness of the 2D Te samples continued to decrease (less than 9.1 nm), degeneracy in the *E*_1_ transverse (TO) and LO modes was observed with peak broadening, this may be caused by the symmetry assignments, electronic band structure changes, and thickness-dependent intra-chain atomic displacement for each band. Meanwhile, due to the unique chiral-chain structure, significant peak shifts were found in the Raman spectra as the thickness varied. Broadband absorption and strong light absorption of few-layer α-Te were investigated by Qiao et al. [64]. The calculated absorbances at 1.6 and 3.2 eV were 2–3% and 6–9% per layer (Fig. 8d, e), respectively, two to three times larger than that of BP. The excellent optical absorbances indicated the promising potential of the few-layer α-Te for broadband optical applications ranging from the visible band to the infrared band. Furthermore, the absorbance showed layer-dependent behavior (Fig. 8f), where the absorption efficiency increased as the sample thickness was reduced. The strong interchain and interlayer couplings in the few-layer α-Te are two key processes that enhance the absorbance significantly. For two incident light sources with wavelengths of 512 and 382 nm, the absorbance of per layer for bilayer α-Te is nearly 1.65 and 2 times higher than that of bulk Te, respectively.

### Thermoelectric Properties

**Fig. 9:**
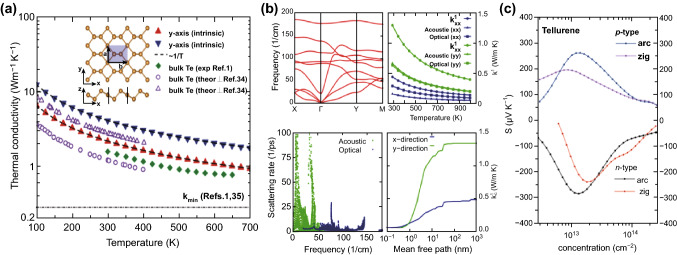
**a** Temperature dependence of the 2D Te lattice thermal conductivity. Adapted with permission from [94]. Copyright 2018, The Royal Society of Chemistry. **b** Phonon band structure, lattice thermal conductivity, scattering rate, and cumulative lattice thermal conductivity, top-left to bottom-right, respectively. Adapted with permission from [95]. Copyright 2018, American Chemical Society. **c** Seebeck coefficient (S) along the armchair and zigzag directions at 300 K. Adapted with permission from [29]. Copyright 2018, The Royal Society of Chemistry

With the increasing global consumption of energy and the shortage of fossil fuel resources, it is of great significance to harvest waste heat energy. Thermoelectric technology provides an effective way to convert the waste heat into useful electricity on a large scale. Since the discovery of 2D materials, they have attracted considerable attention for thermoelectric applications. However, compared to the well-studied thermoelectric properties of the other 2D materials, including graphene, monoelemental borophene [87], germanene [88–90], silicene [88, 91], and arsenene [92, 93], the thermoelectric properties of 2D Te nanoflakes have received relatively little attention. In this section, we highlight some representative investigations into the thermoelectric properties of 2D Te nanoflakes. Gao et al. [94] investigated the thermal properties of 2D Te flakes theoretically by applying first-principles calculations and phonon Boltzmann transport, as shown in Fig. 9a. According to the calculated results, 2D Te possesses an extremely low room-temperature lattice thermal conductivity (*K*_L_) of only 2.16 and 4.08 W m^−1^ K^−1^ along the armchair and zigzag directions, respectively, which are comparable to that of bulk Te. More importantly, compared to the other 2D materials, the calculated *K*_L_ of the 2D Te was the lowest, and this can be attributed to the ultra-low-energy optical modes, soft acoustic modes, and intensive scattering of optical-acoustic phonons. Subsequently, Sharma et al. investigated the thermoelectric properties of the 2D Te by combining first-principles calculations with semi-classical Boltzmann transport theory. The 2D Te was found to possess the lowest K_L_ compared to the other monoelemental 2D materials (Fig. 9b) [95]. This was attributed to the intensive scattering of acoustic phonons into optical phonons. Lin et al. [29] explored the thermoelectric properties of single-layer 2D Te using DFT calculations. Similar to the previous investigations, the anharmonic scattering process dominated and effectively limited its lattice thermal conductivity. Consequently, the calculated *K*_L_ represented the lowest value among the previously investigated monoelemental 2D materials (Fig. 9c).

### Stability

The environmental stability is another important property of 2D materials. For many 2D materials, such as BP [19, 21, 96–98] and TMDCs [99–102], instability has severely hindered their further development in both academic and industrial applications. In sharp contrast, extraordinary environmental stability has been demonstrated for various thicknesses of 2D Te (ranging from few-layer to monolayer). The superior environmental stability is mainly due to existence of an energy barrier in the oxidation pathways, which can effectively prevent the 2D Te being oxidized by environmental oxygen and water. The great environmental stability provides plenty of opportunities for the use of 2D Te nanoflakes in academic and industrial applications, such as high-performance photodetectors and FETs based on air-stable 2D Te. To facilitate a better understanding of the advancements provided by 2D Te, a comparison of the main physical properties of 2D Te and other 2D materials is listed in Table 1.

**Table 1:** Physical properties of 2D Te and other 2D materials

Material	Bandgap (eV)	Room-temperature carrier mobility (cm^2^ V^−1^ s^−1^)	Room-temperature thermal conductivity (W m^−1^ k^−1^)	Environmental stability	Optical absorbance per layer	Refs.
2D Te	0.35–1.265 Direct bandgap	~10^3^	2.16 and 4.08 (armchair and zigzag directions)	~ 2 months	~ 2–3% (774 nm) and ~ 6–9% (388 nm)	[25]
Graphene	0	~ 2.5 × 10^4^	3080–5150	~ 2.5 months	~ 2.3 ± 0.2%	[5, 103]
BP	0.3–1.5 Direct bandgap	~ 10^3^	34 ± 4 and 86 ± 8 (armchair and zigzag direction)	~ 50 h	~ 1–1.5% (774 nm) and 2–3% (388 nm)	[104]
MoS_2_	0.75–1.89 Indirect to direct bandgap	~ 480	101.43 ± 1.13 and 110.30 ± 2.07 (armchair and zigzag direction)	~ 3 months	~ 10% (688 nm) to 30% (442 nm)	[105, 106]
MoSe_2_	0.80–1.58 Indirect to direct bandgap	~ 50	43.88 ± 1.33 and 41.63 ± 0.66 (armchair and zigzag direction)	~ 21 days	~ 10% (775 nm) to 30% (476 nm)	[107, 108]
WS_2_	0.75–1.91 Indirect to direct bandgap	~ 968	32 and 53 (monolayer and bilayer)	~ 2 weeks	~ 10% (620 nm) to 30% (430 nm)	[109, 110]
WSe_2_	0.90 to 1.54 Indirect to direct bandgap	~ 500	3.935	~ 30 days	~ 10% (750 nm) to 30% (430 nm)	[111, 112]

## Applications

Owing to the unique helical chain structure, excellent environmental stability, high carrier mobility, and low-cost synthesis methods of 2D Te nanoflakes, it holds great potential for high-performance 2D material-based electronic and photoelectronic devices. In this section, we summarize some recent representative progress in the applications of 2D Te nanoflakes.

### Photodetector

A photodetector is a device that converts light signals into electrical signals, which is crucial in many fundamental research and practical applications. The strong light-matter interaction, large-scale, and environmental stability of 2D Te nanoflakes make them a promising candidate material for high-performance photodetector applications. In general, the mechanisms of photocurrent generation in 2D Te nanoflakes are photovoltaic, photobolometric, photogating, and photothermoelectric effects [113–115]. The metrics employed to characterize the performance of a photodetector include the specific detectivity, response spectrum range, response time, external quantum efficiency, photogain, noise equivalent power, and photoresponsivity. Here, we discuss the performance of a photodetector based on 2D Te nanoflakes with free-space and waveguide configurations using the metrics.

**Fig. 10:**
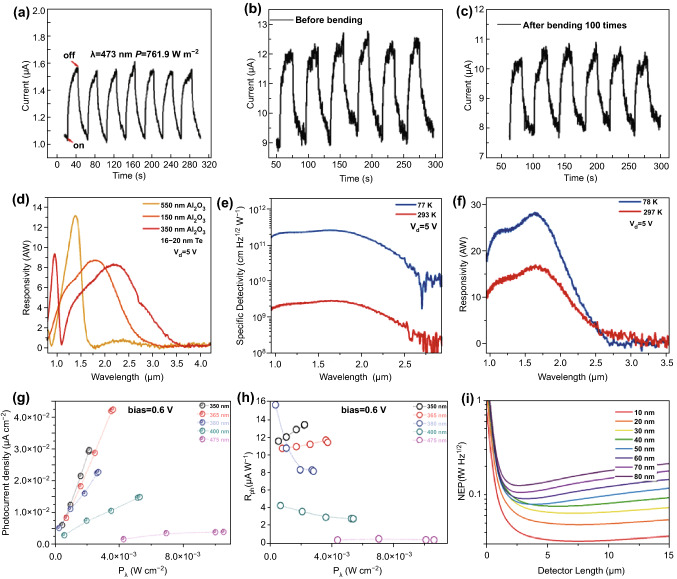
**a** Photoresponse of the 2D Te-based device as a function of time at a bias voltage of 2 V as the laser is switched on and off. **b**, **c** Photoresponse as a function of time while switching the laser on and off, before and after bending the device 100 times, respectively. Adapted with permission from [67]. Copyright 2014, American Chemical Society. **d** Responsivities of devices fabricated on optical cavities with different Al_2_O_3_ thicknesses. **e** Spectral responsivity of a Te photoconductor at 78 and 297 K under a gate bias of *V*_d_ = 5 V. **f** Specific detectivity of Te photoconductors with optimized thickness. Adapted with permission from [26]. Copyright 2018, American Chemical Society. **g**, **h** Photocurrent and photoresponse as a function of incident power P_λ_ for a range of wavelengths. Adapted with permission from [75]. Copyright 2018, Wiley. **i** Calculated NEP of the detector as a function of the 2D Te thickness and device length. Adapted with permission from [137]. Copyright 2019, American Chemical Society

Three of the most well-known and intensively investigated 2D materials are BP [116–120], TMDCs [121–131], and graphene [132–136] due to their superior properties and strong light-matter interactions. As a new member of the 2D materials family, 2D Te has received less attention than the aforementioned three sorts of 2D materials. However, the excellent environmental stability, simple synthesis, high quality, and large achievable scale of 2D Te nanoflakes have recently motivated a surge of academic interest. Subsequently, experiment results have indicated its suitability for high-performance photodetectors. For example, Wang et al. reported a high-photoresponsivity, flexible photodetector based on vdWE-synthesized hexagonal 2D Te nanoplates on a flexible mica substrate [25].The fabricated photodetector exhibited excellent stability and photoresponsivity, as shown in Fig. 10a. The measured current under illumination presented the same level of both noise and photocurrent, and the corresponding photoresponsivity was approximately 162.4 A W^−1^, indicating the high stability and photoresponsivity of the 2D Te-based photodetector. More importantly, the measured photocurrent and noise current only changed slightly after the device was subjected to100 continuous bending cycles (Fig. 10b, c), which proved the device is suitable for wearable and flexible optoelectronic device applications. Subsequently, Amani et al. [26] demonstrated short-wave infrared photodetectors based on solution-synthesized, environmentally stable quasi-2D Te nanofilms. An Au/Al_2_O_3_ optical cavity substrate was employed to further enhance the absorption of the device. Additionally, by adjusting the Al_2_O_3_ spacer thickness, the peak photoresponsivity wavelength of the device can be tuned from 1.4 μm (13 A W^−1^) to 2.4 μm (8 A W^−1^), with nonzero photoresponsivity up to 3.4 μm, as shown in Fig. 10d [26]. In order to further characterize the performance of the fabricated photodetector, the responsivity as a function of various laser wavelengths was measured for device temperatures of 78 and 297 K (Fig. 10e). The responsivity peaked at *λ* = 1.7 μm with values of 27 A W^−1^ (at 78 K) and 16 A W^−1^ (at 297 K). The corresponding calculated specific detectivity at 78 and 297 K were 2.6 × 10^11^ and 2.9 × 10^9^, respectively (Fig. 10f). The enhancement of the specific detectivity at 78 K was due to more efficient suppression of the noise current than at room temperature, which was inversely proportional to the specific detectivity. These outcomes proved that the solution-synthesized 2D Te nanoflakes were suitable for high-performance photodetectors covering the whole near infrared (IR) band. Recently, Xie et al. have demonstrated a high photoresponse photodetector based on LPE-synthesized 2D non-layered Te nanosheets [75]. Photoelectrochemical measurements were taken to evaluate the photoresponse of the fabricated photodetector. In contrast to the previous investigations, this study mainly focused on the photoresponse of the device from the UV to visible bands. At a fixed bias voltage and KOH solution concentration, measurements of photocurrent and photoresponse as a function of incident laser power at different wavelengths were carried out, as shown in Fig. 10g, h, respectively. The measured photocurrent was significantly enhanced by increasing the incident laser power for the five different wavelengths employed in the experiment. Consequently, the photoresponse, which is proportional to the photocurrent, was strengthened as well. Meanwhile, the stability and KOH solution concentration dependence measurements were also taken, and the outcomes indicated that the LPE-synthesized 2D non-layered Te nanosheet is a promising material for photodetectors in the UV to visible bands as well as other photoelectric applications. Due to the small and tunable bandgap of the 2D Te nanoplates, it is a potential material for mid-IR (MIR) photodetector applications. Compared to a free-space detector, waveguide integration can significantly improve the signal-to-noise ratio. The optical absorption behavior was found to be proportional to the path length of the waveguide. Moreover, the detectable bandwidth for waveguide-integrated photodetectors was wider than that of free-space photodetectors, mainly due to the reduced carrier transit time and RC delay. In this regard, Deckoff-Jones et al. [137] have recently reported a waveguide-integrated photodetector based on 2D Te. The low gated carrier concentration and small tunable bandgap of the 2D Te enabled an extremely low-noise photodetector to be achieved at room temperature. Figure 10i presents the calculated noise equivalent power (NEP) of the 2D Te-based photodetector as a function of the 2D Te thickness and device length. The calculated value was far superior to that of the best level previously presented for MIR waveguide-integrated photodetectors. These interesting findings suggest that 2D Te can be considered as a promising material for integrated on-chip MIR detection.

**Fig. 11:**
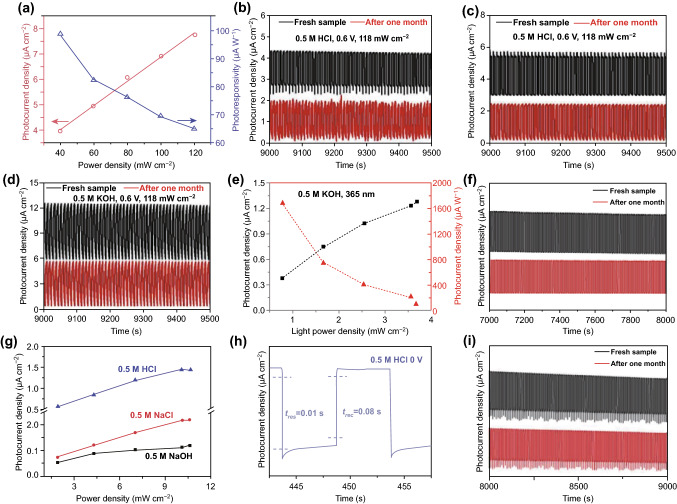
**a** Photocurrent density and photoresponsivity of Te_0.37_@Se_0.63_ as a function of power density in 0.5 M KOH. **b**–**d** Stability measurements of the photocurrent density in different solutions, comparing on/off cycles for a fresh device to a device after 1 month of continuous exposure. Adapted with permission from [138]. Copyright 2019, WILEY–VCH. **e** Photocurrent density and photoresponsivity of the device as a function of incident laser power with a wavelength of 365 nm. **f** Stability measurement of the Te@Bi heterojunction-based photodetector. Adapted with permission from [139]. Copyright 2019. WILEY–VCH. **g** Photocurrent density as a function of the incident laser power with a wavelength of 475 nm in three different electrolytes. **h** Response and recovery time of the Te/Bi_2_Se_3_@Se self-powered device in 0.5 M HCl electrolyte. **i** Stability measurement of the Te/Bi_2_Se_3_@Se self-powered device in 0.5 M HCl electrolyte. Adapted with permission from [140]. Copyright 2019, WILEY–VCH

Recently, photodetectors based on van der Walls heterojunctions composed of the Te nanotubes and bismuth/selenium have been widely investigated due to their significant enhancement of photodetector performance. Huang et al. [138] demonstrated a photoelectrochemical photodetector based on roll-to-roll fabricated Te@Se nanotube heterojunctions for the first time. The heterojunction was synthesized via epitaxial growth of Se on the Te nanotubes. Then, a self-powered photoelectrochemical photodetector based on this heterojunction was fabricated. The photoresponsivity and photocurrent density were found to be significantly enhanced compared to that of the Te nanomaterials alone, as shown in Fig. 11a. Noticeably, the photodetector showed excellent stability under both ambient and harsh conditions, as shown in Fig. 11b–d. Following this study, Zhang et al. [139, 140] demonstrated photodetectors based on Te@Bi and Te/Bi_2_Se_3_@Se heterojunctions. For the Te@Bi heterojunction, the corresponding photocurrent density and photoresponsivity in 0.5 M KOH solution as a function of incident laser power with a wavelength of 365 nm are presented in Fig. 11e. The photocurrent density was approximately proportional to the laser power, while the photoresponsivity was inversely proportional to the laser power. It can be concluded that the generated number of electron–hole pairs was proportional to the incident laser power. Meanwhile, the emergent built-in electric field and plasma effects also have a positive contribution to the photocurrent. The stability measurement was taken in 0.5 M aqueous KOH. The photocurrent density only slightly changed and the device displayed extraordinary stability even after one month of continuous exposure, as shown in Fig. 11f. For the Te/Bi_2_Se_3_@Se heterojunction, the photoelectrochemical photodetector exhibited a wide detection spectrum, ranging from the UV to visible bands. The self-powered photocurrent density measurement is performed in three different solutions (0.5 M HCl, NaCl, and NaOH), as shown in Fig. 11g. The photocurrent density in aqueous HCl was larger than in the NaCl and NaOH solutions, which indicated that the HCl electrolyte was more suitable for the Te/Bi_2_Se_3_@Se-based self-powered photodetector. Furthermore, the response time and stability measurements of the device further confirmed its excellent performance, as shown in Fig. 10 h, i, respectively. Fast response and recovery times of 0.01 and 0.08 s, respectively, were achieved in 0.5 M HCl, which was roughly 50 times faster than that of BP-based devices under same conditions. The photocurrent density of the device in the HCl electrolyte was approximately 90% of the fresh sample value after one month of exposure, demonstrating the extraordinary stability of the device. All these outcomes indicate that the heterojunctions of Te nanotubes and bismuth/selenium have great potential for high-performance photodetector applications. To facilitate a clear comparison, the figures-of-merit for photodetectors based on some typical 2D materials are listed in Table 2. The comparison indicates that 2D Te is suitable for high-performance photodetector applications.

**Table 2:** Typical photodetection performance of devices based on 2D Te and other 2D materials at room temperature

Device	Spectral range (nm)	Room-temperature responsivity	On/off ratio	Mobility (cm^2^ V^−1^ s^−1^)	Specific detectivity (Jones, ${\text{cm}}\,\sqrt {\text{Hz}} /{\text{W}}$)	Rise and decay time	Noise equivalent power	Refs.
Few-layer Te	1400–3500	13 and 8 A W^−1^ at 1400and 2400 nm	10^5^	700	2 × 10^9^ at 1700 nm	1 s and 0.6 ms at 1550 nm	ND	[26]
Few-layer Te	UV–Visible	11–13, 10–11 and 8–16 μA W^−1^ at 350, 365 and 380 nm	10^5^	700	3.1, 2.6, 1.9, and6.8 × 10^7^ at 350, 365, 380, and 400 nm	54.5 and 70.2 ms	ND	[75]
Te_0.37_ and Se_0.63_ heterojunction	UV to near infrared	98.8 μA W^−1^	ND	ND	ND	90 and 100 ms in 0.5 M HCl	ND	[138]
Te and Bi heterojunction	200–800	687 μA W^−1^ at 365 nm in 0.5 M KOH	ND	ND	5.14 × 10^8^	26 and 681 ± 63 ps	ND	[139]
Te/Bi_2_Se_3_ and Se heterojunction	UV to near infrared	170.59μA W^−1^	ND	ND	8.56 × 10^8^	0.72/0.91, 0.66/0.87 and 0.01/0.08 s in 0.5 M NaOH, NaCl, and HCl electrolytes	ND	[140]
Double-layer graphene heterojunction	Visible to 3200	4, 1.9, and 1.1 A W^−1^ at 1300, 2100, and 3200 nm	ND	ND	ND	ND	ND	[141]
Few-layer BP	Visible to 940	4.8 mA W^−1^ at 640 nm	10^5^	1000	ND	1 and 4 ms at 640 nm	ND	[142, 143]
Multilayer b-As_0.83_P_0.17_	2400–8050	15–30 mA W^−1^	ND	ND	> 1.06 × 10^8^	0.54 and 0.52 ms at 4034 nm	< 4.35 pW Hz^−1/2^	[144]
BN/multilayer b-As_0.83_P_0.17_/BN	3400–7700	1.2 mA W^−1^ at 7700 nm	110	1.9 × 10^3^	ND	ND	4300 pW Hz^−1/2^ at 7700 nm	[145]
BP and MoS_2_ heterojunction	1400–3800	0.9 A W^−1^ at 2500 to 3500 nm	10^5^	ND	1.1 × 10^10^ at 3800 nm	3.7 and 4 μs at 2700 nm	ND	[146, 147]
Bilayer MoS_2_	445–1250	5.2 A W^−1^ at 1070 nm	ND	120	ND	400 and 216.5 s	ND	[80, 148]
Multilayer PdSe_2_	450–10,600	42.1 A W^−1^ at 10,600 nm	ND	158	1.1 × 10^9^ at 10,600 nm	74.5 and 93.1 ms at 10,600 nm	< 0.28 pW Hz^−1/2^	[149, 150]
Few-layer WS_2_	457–647	21.2 μA W^−1^ at 568 nm	ND	140	ND	5.3 ms at 514 nm	ND	[151, 152]
Few-layer WSe_2_	370–1064	0.92 A W^−1^	ND	140	ND	0.9 and 2 s at 635 nm	ND	[153, 154]
WSe_2_ and SnS_2_ heterojunction	350–800	244 A W^−1^ at 550 nm	10^7^	0.149	1.29 × 10^13^ at 550 nm	13 and 24 ms at 550 nm	ND	[155

### Field-Effect Transistors

**Fig. 12:**
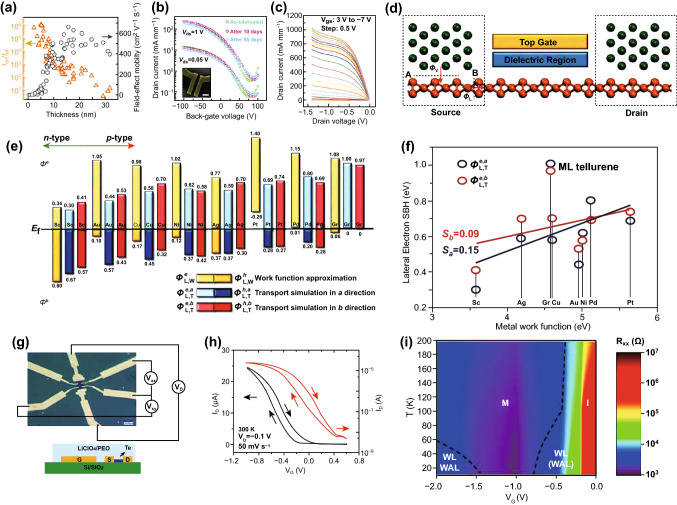
**a** Measured on/off ratio and field-effect mobility of transistors based on 2D Te as a function of thickness. **b** Transfer curves of the 15-nm-thick Te-based transistor. Inset: SEM image of the 2D Te-based transistor. **c** Output curves of the 2D Te-based transistor with a thickness of 11.1 nm and a channel length of 300 nm. Adapted with permission from [25]. Copyright 2018, Nature Publishing Group. **d** Schematic of monolayer Te-based FETs. **e** Lateral Schottky barrier height (SBH) comparison of the monolayer Te-based FET. **f** Lateral electron SBH comparison versus the metal work function in the A and B direction (from part **d**) for the monolayer Te-based FETs. Adapted with permission from [174]. Copyright 2018, The Royal Society of Chemistry. **g** Optical microscopy image and schematic side-view of a 2D Te-based EGT. **h** Transfer curves of the device at 300 K. **i** Gate-voltage-dependent electronic phase diagram of the Te-based EGT. Adapted with permission from [175]. Copyright 2019, American Chemical Society

Transistors are the elementary “building blocks” of integrated circuits, which are used in most modern electronic devices. Since the discovery of graphene and other 2D materials [156–158], such as BP [143, 159–163], and TMDCs [125, 164–168], substantial research interest has been focused on the development of transistors with 2D materials [169–172]. To date, a few high-performance FETs based on 2D materials have been demonstrated. For example, Li [143] and Du [173] et al. reported the first BP-based FETs, in which a field-effect hole mobility of 1000 cm^2^ V^−1^ S^−1^ and an on–off ratio greater than 10^5^ were achieved, which is superior to devices based on TMDCs. However, their environmental instability has severely restricted further development. As previously discussed, 2D Te nanoflakes possess excellent environmental stability, which enables their use in high-performance FETs. Additionally, the unique helical chain structure gives rise to high carrier mobility and strong in-plane anisotropic properties. These superior properties further confirmed the potential of 2D Te nanoflakes in logic electronics applications. Recently, Ye et al. have demonstrated a high-performance FET based on 2D Te nanoflakes produced by the solution synthesis method. With a channel length of 3 μm, the fabricated device showed a large drain current that exceeded 300 mA mm^−1^ and an on/off ratio of approximately 10^5^ [25]. Moreover, field-effect mobilities of approximately 700 cm^2^ V^−1^ s^−1^ were achieved for the optimal 2D Te sample thickness (~ 15 nm) at room temperature (Fig. 12a). Measurements were taken to explore the environmental stability of the device, as shown in Fig. 12b. The drain current only changed slightly after 55 days exposed in air without any encapsulation treatment, demonstrating the excellent air-stability of the 2D Te nanoflakes. A highest drain current exceeding 1.06 A mm^−1^ was obtained by further reducing the channel length, representing the largest value among all 2D material-based transistors and comparable to that of conventional semiconductor devices (Fig. 12c). These outcomes indicate the great potential of 2D Te nanoflakes in high-performance electronic and photoelectronic applications. Yan et al. [174] also reported the first comprehensive simulation of the interfacial characteristics of monolayer 2D Te with various metals and 2D graphene electrodes based on quantum transport simulation and ab initio electronic structure calculations, as shown in Fig. 12d. According to their investigation, a lateral n-type Schottky contact was formed with the Au and Sc electrodes in both directions, respectively. For electrodes of other metals, such as Cu, Ag, Pd, Pt, and Ni, a lateral p-type Schottky contact was formed in both directions, as shown in Fig. 12e. The formation of the Schottky barrier was primarily caused by the strong Fermi level pinning effect (Fig. 12f). For the 2D graphene electrode, a lateral p-type Ohmic contact was formed in both directions, which was caused by the combination of a weak Fermi level pinning effect at the interface and the work function match of monolayer graphene with the VBM of the monolayer 2D Te. Consequently, 2D graphene is the most promising electrode material for FETs based on monolayer 2D Te. Ren et al. reported high-performance electrolyte-gated transistors (EGTs) based on solution-grown 2D Te nanoflakes (Fig. 12g), and a gate-tuned insulator–metal transition was observed at low temperature [175]. By using Hall effect measurement, the fabricated p-type EGTs exhibited charge densities exceeding 10^13^ cm^−2^, mobilities greater than 400 cm^2^ V^−1^ S^−1^, and an operating voltage less than 2 V. Additionally, resistance–temperature measurements were taken to reveal the transport mechanisms. Meanwhile, a 2D insulator–metal transition was formed with a charge density of 1.6 × 10^13^ cm^−2^ at the surface of the 2D Te (Fig. 12i). These outcomes indicate that electrolyte gating is an effective means of modifying the charge density-dependent properties of 2D Te nanoflakes.

**Fig. 13:**
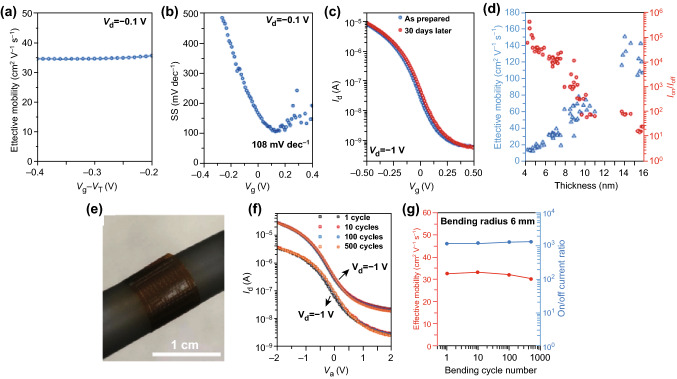
**a**, **b** Effective mobility and subthreshold swing of Te-based (8 nm) FETs. **c** Drain current and gate voltage (*I*_d_–*V*_g_) measurement of Te-based (8 nm) FETs with a time period of 30 days. **d** Thickness dependence of the on/off current ratio (red) and effective mobility (blue) for Te-based FETs. **e** Optical image of Te-based FETs on a Kapton substrate. **f**, **g** Electrical properties of the device after 500 bending cycles with a radius of 6 mm. Adapted with permission from [178]. Copyright 2020, Nature publishing Group. (Color figure online)

Recently, Zhao et al. have demonstrated high-performance wafer-scale p-type FETs based on high-quality 2D Te thin films with a thickness of 8 nm synthesized by thermal evaporation. The effective hole mobility, on/off current ratio, and subthreshold swing were measured to be 35 cm^2^ V^−1^ S^−1^, 10^4^, and 108 mV dec^−1^, respectively (Fig. 13a, b). Additionally, the fabricated device displayed extraordinary environmental stability even after exposure to ambient conditions without any encapsulation for 30 days, as shown in Fig. 13c. The dependence of the effective mobility and on/off current ratio on the Te nanoflake thickness was also investigated to further evaluate the performance of Te-based FETs, as shown in Fig. 13d. The effective mobility was proportional to the thickness of the Te, which is due to the reduced effect of surface roughness scattering for thicker films [176, 177]. However, the on/off current ratio decreased monotonically as the Te sample thickness increased, which can be attributed to the decreased bandgap of the Te channel. Electrostatic control was also suppressed as the thickness of the Te nanoflakes increased. The Te nanoflakes were synthesized via low-temperature evaporation, which is beneficial for depositing Te on various substrates, including plastic and glass. Thus, FETs based on Te synthesized by low-temperature evaporation hold great potential for flexible and transparent electronics and display applications. Motivated by these results, Te-based FETs were fabricated on a Kapton substrate to evaluate their mechanical flexibility and operational stability, as shown in Fig. 13e. The electrical properties of the device only changed slightly even after 500 bending cycles with a radius of 6 mm, as shown in Fig. 13f, g, indicating the extraordinary resilience of Te-based FETs in flexible applications.

### Piezoelectric Devices

**Fig. 14:**
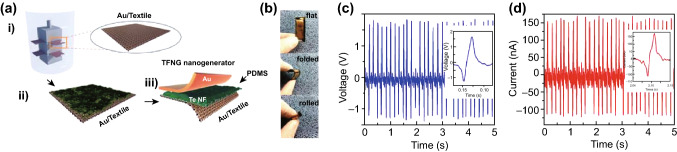
**a** Schematic of the tellurene flexible nanogenerator (TFNG) device fabrication process. **b** Optical images of the TFNG devices in different states: flat, folded, and rolled. **c**, **d** Measurements of the output voltage and current due to periodic bending and unbending behavior. The insets show a single bending–unbending event. Adapted with permission from [183]. Copyright 2016, Elsevier

Owing to the superior piezoelectric and mechanical performance, and nanoscale structure, the piezoelectric effect in 2D materials has attracted considerable attention due to the potential applications, including energy converters, energy sources, sensors, and actuators. For example, due to the inversion asymmetry of monolayer MoS_2_, both experimental and theoretical investigations have proved that monolayer MoS_2_ exhibits piezoelectricity in its 2H phase [179–181]. However, the conversion rate of mechanical to electrical energy is severely restricted by its small piezoelectric coefficient, which also limits its practical applications. In sharp contrast, 2D Te nanoflakes possess a large work function and the largest piezoelectric strain coefficient compared to the other existing piezoelectric materials [182]. Consequently, it has been a promising candidate for application in nanogenerators. Recently, He et al. [183] have demonstrated the first fully wearable and flexible nanogenerators with high output power based on low-temperature, hydrothermally synthesized 2D Te nanoflakes. The fabricated 2D Te nanogenerator device was composed of a sandwich-like structure with polydimethylsiloxane (PDMS)-coated Te nanoflakes and Au/textile material, with the Au layer employed as the top and bottom electrodes. The devices were investigated in three bending states: flat, folded, and rolled, as shown in Fig. 14a, b. Under identical strains, periodic bending tests were carried out, and a closed-circuit current and an open-circuit voltage of 290 nA and 3 V were achieved, respectively (Fig. 14c, d). In order to confirm the potential of the fabricated 2D Te nanogenerator device for converting vibrational energy from human activities into electrical energy, the device was adhered to a human arm, and the corresponding output current and voltage due to periodic bending and unbending were measured to be 650 nA and 2.5 V, respectively. Additionally, when a compressive force of 8 N and an increased driving frequency of 10 Hz were applied to the device, the output power density was as high as 2.07 mW cm^−2^, which can power at least 10 LEDs. In order to get a better insight into the piezoelectric device based on 2D Te nanoflakes, Chen et al. carried out a systematic investigation of 2D Janus tellurene (Te_2_Se), including its piezoelectric properties and stability for monolayer and multilayer based on first-principles calculations. According to the calculation for the monolayer 2D Janus tellurene, the flexible mechanical properties and structural symmetry-breaking give rise to large in-plane and out-of-plane piezoelectric coefficients of 16.28 and 0.24 pm V^−1^, respectively. For multilayer 2D Janus tellurene, the applied in- and out-of-plane strains give rise to strong piezoelectric effects. Furthermore, certain stacking sequences lead to out-of-plane piezoelectric effects, while other sequences produced an in-plane piezoelectric effect. Remarkably, the calculated piezoelectric coefficients of monolayer and multilayer 2D Janus tellurene were larger than that of the many Janus TMDCs and other well-known piezoelectric materials.

### Modulator

**Fig. 15:**
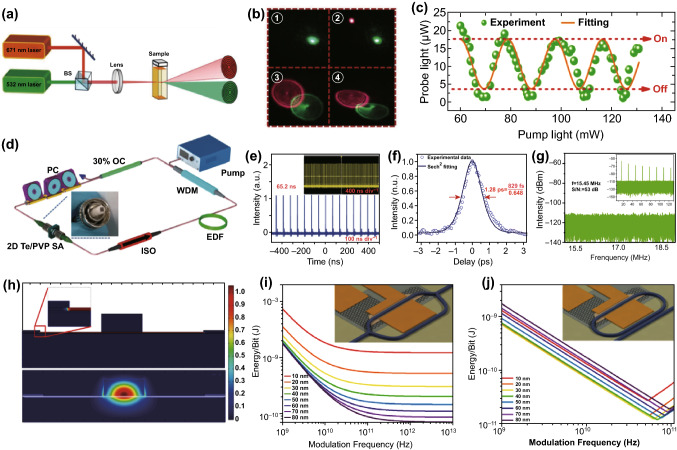
**a** Schematic of the all-optical modulator system based on 2D Te nanoflakes. **b** Diffraction rings produced by the all-optical modulator system. **c** Output of the all-optical switcher based on 2D Te nanoflakes as a function of pump laser power including the theoretical fit. Adapted with permission from [184]. Copyright 2019, WILEY–VCH. **d** Schematic of the mode-locking erbium-doped fiber laser based on a 2D Te/PVP thin film. **e** Pulse trains from the fiber laser. Inset: 4 μs pulse trains. **f** Pulse duration of the modulated femtosecond laser. **g** Radio-frequency spectrum of the fiber laser. Inset: broadband radio-frequency spectrum. Adapted with permission from [185]. Copyright 2019, The Royal Society of Chemistry. **h** Calculated electric and optical field profiles in the modulator. **i** Simulated energy consumption of the modulator as a function of frequency for different thicknesses of 2D Te nanoflakes. Inset: schematic of the Mach–Zehnder interferometer (MZI) modulator. **j** Calculated energy consumption of the Te racetrack modulator as a function of frequency for different thicknesses of tellurene. Inset: schematic of the racetrack modulator. Adapted with permission from [137]. Copyright 2019, American Chemical Society

The superior properties of 2D Te nanoflakes, such as broadband optical absorption and response, strong light-material interaction, and excellent environmental stability, enable 2D Te nanoflakes to be utilized for optical modulators. To gain insight into the modulation mechanism, Wu et al. designed an all-optical modulation system based on 2D Te nanoflakes, with “on” and “off” modes for the modulating behavior [184]. The schematic of the system with 2D Te nanoflakes dispersions based on spatial cross-phase modulation is shown in Fig. 15a. Due to the combination of the Kerr and non-axis-symmetric thermal convection effects for both the pump and probe light, the diffraction rings were distorted, as seen in Fig. 15b. Moreover, the lower-power probe light can be modulated by higher-power pump light by using this modulation system, and the “on” and “off” modes can be realized (Fig. 15c). These outcomes show the 2D Te nanoflakes to be a promising candidate for photonics device applications. Motivated by the superior properties and great potential of 2D Te nanoflakes for modulator applications, Guo et al. successfully fabricated a saturable absorber based on 2D Te/PVP nanoflakes, enabling the achievement of a highly stable femtosecond laser with a pulse duration of 829 fs (Fig. 15f) [185]. The 2D Te/PVP thin film was adhered to the end of a fiber and employed as a saturable absorber (Fig. 15d). The generated pulse train is shown in Fig. 15e, with a repetition frequency of 15.45 MHz. Moreover, no peak intensity fluctuation was found in the pulse train, illustrating the excellent stability of the mode-locked laser operation. To further investigate the stability of the mode-locked laser, radio-frequency (RF) spectrum measurement was carried out, as shown in Fig. 15g. The peak was measured, displaying a frequency and peak-to-background ratio of 15.45 MHz and 53 dB, respectively. These outcomes further confirmed the extraordinary stability of the mode-locked laser operation. Furthermore, the saturation intensities (modulation depths) of the Te/PVP thin films were measured to be 44.65 (11.86%), 26 (10.5%), and 78.14 (27%) GW cm^−2^ at 800, 1060, and 1550 nm, respectively, which confirmed that Te/PVP thin films show great potential for broadband saturable absorber and mode-locking laser applications. As mentioned previously, similar to BP, 2D Te has a tunable bandgap range of 0.35–1.2 eV, which covers the MIR spectral band up to a wavelength of 3.5 μm. Several kinds of MIR modulators have been demonstrated using free carrier plasma dispersion [186, 187], thermo-optic phase shift [188–190], electro-absorption (Pauli blocking or field-induced effects) [191–193], and the electro-refractive (Pockels) effect [194]. Among these technologies, Pockels electro-optic modulators were the most popular modulator due to their intrinsic ultrafast response and potential for achieving phase-only modulation. However, MIR integrated Pockels modulators have only been experimentally realized using Si-on-LiNbO_3_ [194]. The broken structural inversion symmetry and huge electro-optic activity of 2D Te permit it to be utilized for low energy and ultrafast Pockels effect modulators. In this regard, Jones et al. reported a high-performance waveguide-integrated Pockels effect modulator based on 2D Te. The modulator showed a switching energy of 12.0 pJ/bit and a half-wave voltage-length product of 2.7 V cm, which is orders of magnitude higher than that of existing state-of-the-art devices (Fig. 15h–j) [137].

### Energy Harvesting Devices

**Fig. 16:**
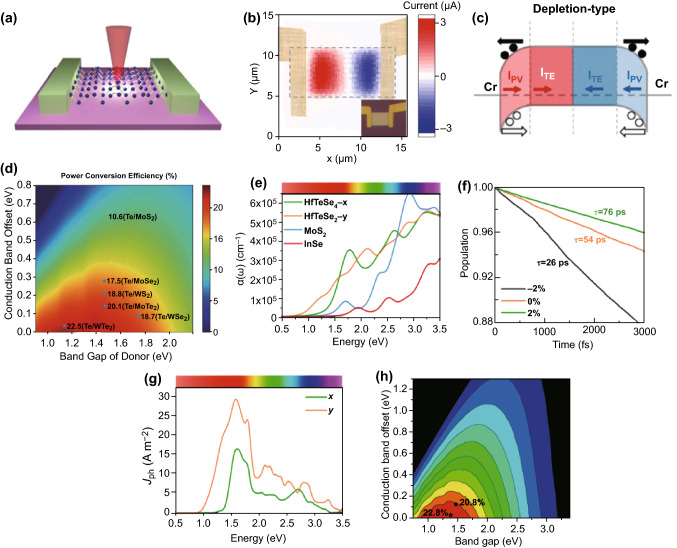
**a** Schematic of the thermoelectric device using 2D Te. **b** Laser-induced thermoelectric current mapping of the device. **c** Band diagrams of the depletion-type device due to the combined photoelectric and thermoelectric effects. Adapted with permission from [202]. Copyright 2019, American Chemical Society. **d** Power conversion efficiency of 2D Te/TMDCs heterojunction solar cells as a function of the conduction band offset and the donor bandgap. Adapted with permission from [209]. Copyright 2019, The Royal Society of Chemistry. **e** Absorption coefficients of monolayer HfTeSe_4_ compared to MoS_2_ and InSe. **f** Carrier recombination lifetime under different biaxial strains. **g** Photocurrents of monolayer HfTeSe_4_ as a function of photon energy along the x- and y-direction. **h** Power conversion efficiency of the HfTeSe_4_/Bi_2_WO_6_ heterostructure-based solar cell with and without a 2% tensile strain. Adapted with permission from [210]. Copyright 2019, American Chemical Society

Due to the rapid pace of industrial development, the energy crisis has become a critical issue in the twenty-first century. During the past decade, there have been tremendous efforts to solve this severe worldwide problem [195–200]. The generation of electricity through harvesting energy from ambient and waste heat sources is an environmentally friendly and sustainable approach to overcome this problem. In general, two solutions have been proposed to improve thermoelectric generation efficiency: striving to develop high-efficiency thermoelectric bulk materials or low-dimension thermoelectric materials. As previously mentioned, a large body of theoretical and experimental investigations has proved that 2D Te possesses extraordinary thermoelectric properties, even among the other 2D materials [185, 201–205]. Thus, 2D Te holds great potential for next-generation thermoelectric device applications. Recently, by combining the advantages of nanostructures and the intrinsically high thermoelectric property of 2D Te, Qiu et al. [202] have presented the first highly efficient thermoelectric devices based on 2D Te, as shown in Fig. 16a. A He–Ne laser was employed to locally heat the 2D Te and generate a temperature gradient. Thermoelectric current mapping measurements were taken to further improve the harvesting efficiency. Figure 16b presents the laser-induced thermoelectric current mapping of the fabricated device. Noticeably, a thermoelectric current of almost 3 μA was achieved for an incident laser power of 3 mW, which is two orders of magnitude larger than that of previous investigations [206, 207]. However, a photovoltaic effect may exist during the measurement and contribute to the generated current. To evaluate of the influence of photovoltaic effect during the measurement process, three different types of metals were used as electrodes. According to the outcomes, the photovoltaic effect generated a current located at the metal–semiconductor interface, and a depletion-type contact was formed, as shown in Fig. 16c. Solar energy is another important reliable source of energy in nature. Many efforts have attempted to efficiently take advantage of this energy source. Solar cells, which can convert sunlight into electricity, have already proven to be a lucrative candidate for commercialization applications and continue to be an extremely popular and diverse area of research. To further enhance the conversion efficiency and performance of solar cells, heterojunction solar cells are currently in the research and development phase. This type of solar cell requires a suitable direct bandgap of 1.2–1.6 eV, high carrier mobility, and environmental stability. It has been shown that 2D tellurene meets almost all the aforementioned criteria [208]. Recently, Wu et al. [209] have theoretically demonstrated a high-efficiency heterojunction solar cell based on 2D Te and TMDCs. By utilizing first-principles DFT simulations, the maximum power conversion efficiency of the 2D Te/WTe_2_ and 2D Te/MoTe_2_ heterojunction solar cells was calculated to be 22.5% and 20.1%, respectively, as shown in Fig. 16d. In addition, the heterojunctions exhibited a remarkable absorption of sunlight and an enhancement of charge separation behavior due to the type-II band alignment. Utilizing pure 2D ternary compounds, Yang et al. [210] reported a highly efficient solar cell based on monolayer HfTeSe_4_. The simulation outcomes from first-principles calculations indicated that the solar cell exhibited an extraordinary absorbance coefficient of up to 10^5^ cm^−1^ in the visible band, as shown in Fig. 16e. The monolayer HfTeSe_4_ exhibited a relative long carrier recombination lifetime and ultrahigh photocurrent (Fig. 16f, g), which is beneficial for solar cell applications. The calculated maximum power conversion efficiency of solar cell based on a monolayer HfTeSe_4_ and Bi_2_WO_6_ heterojunction is up to 20.8% (Fig. 16h), which is much higher than that of 2D organic and heterostructure-based solar cells reported previously [211–214]. To aid in the comparison with existing devices, the figures-of-merit of some typical energy harvesting devices based on 2D materials are listed in Table 3. The comparison results indicate that 2D Te is suitable for high-performance energy harvesting devices.

**Table 3:** Typical energy harvesting device performance based on 2D Te and other 2D materials

Material	Thermoelectric figure of merit: ZT (room temperature)	Room-temperature power factor	Power conversion efficiency	Absorbance coefficient (cm^−1^)	Refs.
Few-layer Te	0.63	31.7 mW/cm K^2^	ND	ND	[202]
Monolayer HfTeSe_4_ and Bi_2_WO_6_ heterojunction	ND	ND	20.8%	6 × 10^5^	[210]
2D Te/WTe_2_ and 2D Te/MoTe_2_ heterojunction	ND	ND	22.5% and 20.1%	5 × 10^5^	[209]
Graphene	0.42	2.5 mW/m K^2^	12.6%	3.01 × 10^5^	[215–217]
BP	0. 25	138.9 μW/cm K^2^	6.85%	ND	[218–220]
Perovskite	0.13	28–36 μW/cm K^2^	17.2%	~ 10^4^	[221–223]

### Logic Gates and Circuits

**Fig. 17:**
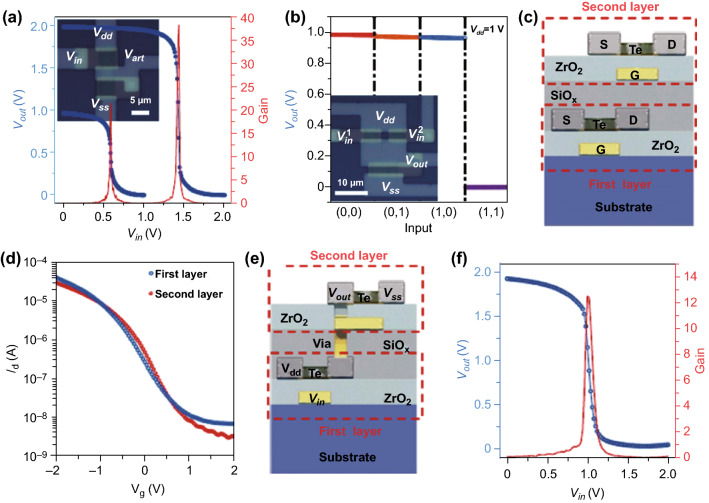
**a**, **b** Inverter, NAND logic gate, and optical images based on p-type Te FETs. **c** Schematic diagram of the 3D multilayer transistors and logic gates. **d** *I*_d_–*V*g transfer curves of the device. **e** Schematic diagram of the 3D circuits. **f** Voltage transfer characteristic of the device. Adapted with permission from [178]. Copyright 2020, Nature Publishing Group

Benefiting from the high uniformity of Te FETs, which enables the fabrication of logic gates and computational circuits based on Te FETs, Javey et al. have recently demonstrated various functional logic gates and circuits based on p-type Te FETs. Firstly, a simple logic gate consisting of two Te p-type FETs and a NAND gate with a logically valid output were fabricated, as shown in Fig. 17a, b. For the logic gate, typical voltage transfer curves with a gain of 22 and 38 (*V*_dd_ = 1 and 2 V) were achieved. Additionally, multiplier circuits consisting of 35 and 39 transistors were fabricated to realize multiplication functions. By increasing the number of transistors, the maximum output voltage loss decreased from 6 to 3%. To further explore the performance of Te in logic gate and circuit applications, more complicated 3D multilayer transistors and logic gates based on p-type Te FETs were demonstrated, as shown in Fig. 17c. Noticeably, the *I*_d_–*V*_g_ transfer curves of the first layer changed slightly after the construction of the top layer through a low-temperature process, as shown in Fig. 17d. The slight shift of the threshold voltage was likely caused by the semiconductor–oxide interface or a fixed charge in the intermediate oxide. A two-layer invert was employed to construct 3D circuits, where the upper- and bottom-layer transistors were operated as an active load and the driver, respectively (Fig. 17e). A gain of approximately 12 at a *V*_dd_ = 2 V was achieved, as shown in Fig. 17f. These outcomes indicate that p-type Te FETs possess great potential for integrated 3D logic gates and circuits applications [178].

## Summary

Since 2D Te nanoflakes were successfully fabricated in 2017, it has become one of the most popular of the 2D materials family [183]. In this review, we summarized the crystal structure, synthesis methods, physical properties, and various applications based on 2D Te nanoflakes, such as photodetectors, FETs, piezoelectric devices, modulators, logic gates, and circuits. Similar to BP, 2D Te nanoflakes are a layered semiconductor material with a thickness-dependent bandgap ranging from 0.35 to 1.2 eV (visible to MIR band). The unique helical chain structure of 2D Te gives rise to a high room-temperature carrier mobility (~ 10^3^ cm^2^ V^−1^ s^−1^) and strong in-plane anisotropic properties. In sharp contrast, due to an energy barrier that inhibits oxidation pathways, 2D Te nanoflakes possess more robust environmental stability than other existing 2D materials, making them a promising material for fundamental research as well as practical applications. During the past two decades, it has become highly sought after to obtain large-scale and high-quality 2D materials to satisfy the demands of various applications arising from the rapid development of the semiconductor industry. Up scaling the fabrication of these 2D materials is currently a major area of focus in the nanotechnology and nanoscience field, and the lack of commercially viable solutions is also severely restricting the further development of 2D materials in the semiconductor industry. However, scalable and high-quality 2D Te nanoflakes can be efficiently synthesized though various means, including PVD, MBE, solution synthesis, LPE methods, and thermal evaporation. These low-cost and efficient synthesis methods are favorable for industry applications and commercialization.

As a versatile material, 2D Te nanoflakes have been utilized in a wide range of applications. The unique helical chain structure, flexible mechanical properties, and structural symmetry-breaking in 2D Te nanoflakes lead to a large in-plane piezoelectric coefficient, which enable it to be a potential material for piezoelectric devices. Moreover, benefiting from air-stability, strong light-material interaction, broadband optical absorption and response, and other superior properties, 2D Te nanoflakes have been utilized in the fabrication of numerous devices, including photodetectors, FETs, modulators, logic gates and circuits, and exhibited excellent performance that often exceeds the existing state-of-the-art 2D materials.

## Prospective

**Fig. 18:**
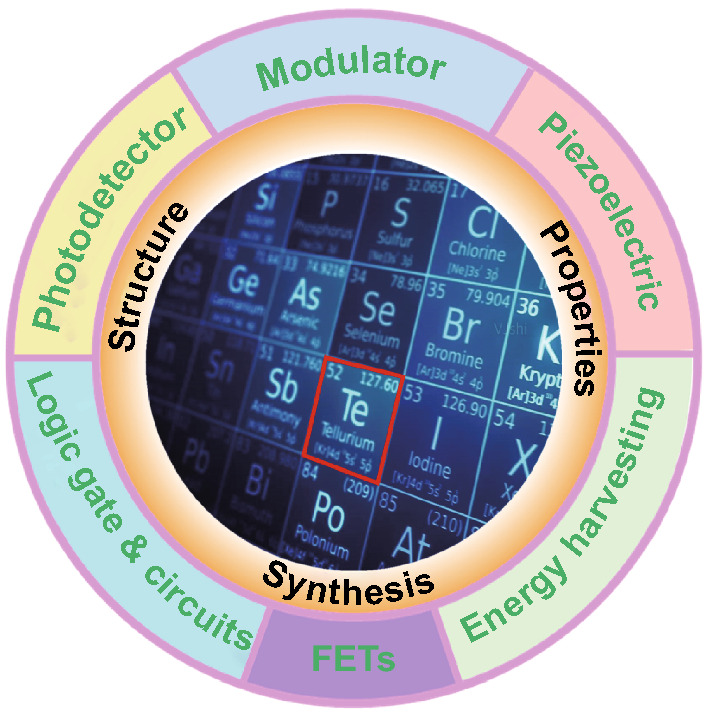
The academic and engineering applications of 2D Te nanoflakes

Although 2D Te nanoflakes have already shown excellent potential for academic and engineering applications (Fig. 18), challenges and opportunities still remain for researchers. For synthesis processes, ultrathin 2D Te nanoflakes can be synthesized through the PVD method. However, the requirements for a high-vacuum environment and high-purity atomic sources limit the potential for up scaling. The LPE technique is an effective means of synthesizing layered 2D Te nanoarchitectures. However, the small-scale and inefficient control of the thickness of the derived materials restricts its further applications. Additionally, in situ CVD methods have been applied to synthesize large-scale and high-quality 2D materials, such as graphene, BP, and TMDCs among others [224–233]. However, the use of CVD method is rarely reported to produce ultrathin 2D Te nanoflakes. Thus, it is of great significance to develop CVD techniques to grow atomically thin Te. In particular, the controllable synthesis of a desired number of layers from monolayer to multilayer is highly sought after.

Regarding photodetectors and FETs based on 2D Te nanoflakes, the reported investigations mainly focus on the visible to near-IR band. The development of ultra-broadband (UV to MIR band) devices is highly preferred. Additionally, the carrier dynamics and transport mechanisms in photodetectors and FETs are not understood clearly enough compared to photodetectors and FETs based on BP and TMDCs. Furthermore, doping and heterojunction methods can significantly enhance the performance of photodetectors and FETs based on 2D Te nanoflakes. To further improve the performance of Te-based devices, it is necessary to improve the doping and heterojunction fabrication techniques, particularly controllable doping and heterojunction formation processes. Further investigations are necessary to gain a better insight into photodetectors and FETs based on 2D Te nanoflakes. In the modulator application, the long-term operation stability is still a challenge. Additionally, only a few investigations have been reported examining free-space and waveguide modulators based on 2D Te nanoflakes. Further research is needed to explore the performance and mechanisms of these modulators. For energy harvesting devices, most investigations are still in the theoretical stage and further experimental work is needed to provide a pathway toward helping solve the energy crisis. In logic gate applications, the use of 2D Te has only been achieved in the simplest logic gate. Further investigations should be carried out to explore the performance of p-type Te FET-based logic gates with more complicated structures. For circuit applications, the practicality of p-type Te FETs has only been demonstrated for monolithic 3D circuits. To enhance the performance of such devices, a more suitable insulation layer and a more optimized deposition technique must be found.

Besides the aforementioned applications, extending the applications of 2D Te thin films to other fields, such as flexible and transparent electronics and displays, highly integrated chips, biomedicine, and lasing has become a critical issue for its further development. With the rapid development of 2D materials and industry demands, we believe 2D Te will continue to find novel applications in the future.

In conclusion, 2D Te is a fascinating material due to its excellent properties and great potential in various fundamental and practical applications. However, 2D Te also faces some significant challenges. The continued investigation of this interesting material in photonic systems, including photodetectors, FETs, piezoelectric devices, modulators, energy harvesting devices, logic gates, and circuits, is anticipated. A more comprehensive understanding of 2D Te nanoflakes will emerge in the future as a result of these ongoing concerted research efforts.
